# Leveraging Classifier Performance Using Heuristic Optimization for Detecting Cardiovascular Disease from PPG Signals

**DOI:** 10.3390/diagnostics14202287

**Published:** 2024-10-14

**Authors:** Sivamani Palanisamy, Harikumar Rajaguru

**Affiliations:** 1 Department of Electronics and Communication Engineering, Jansons Institute of Technology, Coimbatore 641659, India; psiva.bit@gmail.com; 2 Department of Electronics and Communication Engineering, Bannari Amman Institute of Technology, Sathyamangalam 638401, India

**Keywords:** CVD, dimensionality reduction, LR, KNN, LDA, SVM

## Abstract

Background/Objectives: Photoplethysmography (PPG) signals, which measure blood volume changes through light absorption, are increasingly used for non-invasive cardiovascular disease (CVD) detection. Analyzing PPG signals can help identify irregular heart patterns and other indicators of CVD. Methods: This research involves a total of 41 subjects sourced from the CapnoBase database, consisting of 21 normal subjects and 20 CVD cases. In the initial stage, heuristic optimization algorithms, such as ABC-PSO, the Cuckoo Search algorithm (CSA), and the Dragonfly algorithm (DFA), were applied to reduce the dimension of the PPG data. Next, these Dimensionally Reduced (DR) PPG data are then fed into various classifiers such as Linear Regression (LR), Linear Regression with Bayesian Linear Discriminant Classifier (LR-BLDC), K-Nearest Neighbors (KNN), PCA-Firefly, Linear Discriminant Analysis (LDA), Kernel LDA (KLDA), Probabilistic LDA (ProbLDA), SVM-Linear, SVM-Polynomial, and SVM-RBF, to identify CVD. Classifier performance is evaluated using Accuracy, Kappa, MCC, F1 Score, Good Detection Rate (GDR), Error rate, and Jaccard Index (JI). Results: The SVM-RBF classifier for ABC PSO dimensionality reduced values outperforms other classifiers, achieving the highest accuracy of 95.12% along with the minimum error rate of 4.88%. In addition to that, it provides an MCC and kappa value of 0.90, a GDR and F1 score of 95%, and a Jaccard Index of 90.48%. Conclusions: This study demonstrated that heuristic-based optimization and machine learning classification of PPG signals are highly effective for the non-invasive detection of cardiovascular disease.

## 1. Introduction

Cardiovascular disease continues to be the leading cause of death globally, significantly burdening healthcare systems and economies. It accounts for approximately 17.9 million deaths annually, necessitating urgent advancements in prevention, diagnosis, and treatment strategies. Globally, life expectancy is on the rise due to significant advancements in healthcare, medicine, and a heightened awareness of personal hygiene and environmental hygiene [1]. Optimizing computer-aided diagnosis holds immense potential for healthcare. It fosters a more objective and consistent diagnostic approach, ultimately benefiting patient outcomes. Photoplethysmography (PPG) technology has the potential to revolutionize early detection of cardiovascular issues, aimed at increasing life expectancy and reducing healthcare costs [2,3]. It can be easily incorporated into wearable medical technology to measure various health-related metrics. Due to its diagnostic capabilities, PPG is widely utilized in clinical practices. Over the past decades, there has been a swift increase in the implementation of Wireless Patient Monitoring (WPM) and Internet of Things (IoT) models worldwide [4]. Cutting-edge smart home automation and e-healthcare system technologies now enable in-home medical services, reducing the need for hospital visits [5]. Digital health has increasingly been integrated into daily life through digital health tools like smartwatches and health apps, facilitating real-time monitoring and diagnostics [6]. High blood pressure is a significant threat to developing cardiovascular diseases (CVDs) [7]. The World Health Organization (WHO) identifies cardiovascular disease as the leading chronic illness globally, significantly contributing to the overall disease burden and responsible for 31% of deaths worldwide [8]. PPG technology, highlighted as versatile and cost-effective [9], utilizes a photoelectric sensor to detect changes in light transmitted or reflected by blood vessels in the skin [10]. PPG does not require specific placement of sensors at predetermined locations on the body [11]; Measurements can be easily taken from the finger, wrist, or even earlobe [12]. This user-friendly approach makes PPG a popular bio signal for wearables, especially for monitoring heart rate during exercise and physical activity. 

One of the most widely used technologies for monitoring a patient’s physiological conditions is PPG. It is popular due to its non-invasive nature and low cost. Additionally, PPG’s ability to provide continuous readings makes it ideal for use in pulse oximetry [13]. PPG signals require minimal hardware compared to traditional ECG monitoring systems, making them more accessible [14]. Shintomi et al. [15] investigated the effectiveness of compensating for heartbeat errors in mobile and wearable sensor data to improve heart rate variability (HRV) analysis. PPG sensors do not require a reference signal, making them ideal for integration into wristbands. This enhances their application, utility, and clinical applicability, making them highly effective for various research purposes in the analysis and diagnosis of CVD [16]. Moshawrab et al. [17] explored the use of smart wearable devices for detecting and predicting CVDs. The authors also reviewed the development and use of these wearables, which demonstrated their high effectiveness in managing CVDs. Reisner et al. [18] developed the use of the PPG for circulatory monitoring.

PPG signals are mostly preferred for CVD analysis because they are non-invasive and inexpensive while still offering valuable insights into blood flow variations that might indicate cardiovascular issues. In recent years, machine learning algorithms have gained significant attention in medical diagnostics, providing a lot of potential for accuracy and efficiency in the detection of diseases [19]. When paired with effective dimensionality reduction techniques, they can greatly improve the accuracy of CVD diagnosis. This research seeks to address these challenges by exploring the effectiveness of dimensionality reduction techniques in enhancing the accuracy of CVD detection using PPG data. We explore three heuristic DR techniques: ABC-PSO, the Cuckoo Search algorithm (CSA), and the Dragonfly algorithm (DFA). In this work, these techniques are integrated with various classification methods to create a robust framework for early and accurate CVD detection.

Researchers have explored a diverse range of techniques for diagnosing CVD from PPG signals. Several DR techniques can be applied to the PPG dataset to reduce both the dimensionality and computational complexity of PPG signals for CVD classification. The methods used for various types of CVD classification problems, along with their limitations, are presented in Table 1. In light of these limitations, this paper explores advanced techniques such as heuristic optimization algorithms for reducing the dimensionality of PPG data.

Compared to the feature extraction techniques mentioned in Table 1, the selected techniques (ABC-PSO, Cuckoo Search, and Dragonfly) are simpler and provide enhanced interpretability. These techniques allow researchers to identify which features are most significant for classification. This is essential in a medical context, where understanding the reasoning behind a model’s decisions is crucial. Additionally, the selected techniques are typically less computationally intensive than other models. This enables quicker training and analysis, which can be advantageous in a clinical environment.

This study focuses on comparing optimization techniques and classification methods rather than aiming for the absolute highest accuracy. The selected techniques offer a solid basis for this comparison while ensuring interpretability and efficiency. However, it is still important to consider interpretability and data size limitations when applying machine learning algorithms for CVD classification. In summary, the chosen techniques emphasize interpretability, efficiency, and the ability to facilitate comparisons between optimization technique and classification methods, making them well-suited for this particular study.

This research work makes the following key contributions:

The study proposes an early detection and intervention method for cardiovascular diseases using PPG signals.

Three metaheuristic optimization algorithms are used as DR techniques to reduce the dimension of the high-dimensional PPG data.

The dimensionality-reduced PPG data were then analyzed using ten different classification algorithms to detect the presence of CVD. The classifiers’ performance is evaluated using parameters such as accuracy, GDR, MCC, Kappa, error rate, F1 score, and Jaccard index.

In summary, to the best of our knowledge, no study in the literature has exclusively analyzed heuristic-based DR techniques to enhance classifier performance for detecting CVD from PPG data.

The structure of this article is as follows: Section 1 presents the introduction, followed by the methodology in Section 2. Heuristic dimensionality reduction techniques are discussed in Section 3. Section 4 covers the ten different classifiers based on learning and selection parameters used to differentiate between normal and CVD-related segments within the PPG signal data. The findings of the work are thoroughly examined and interpreted in Section 5, and the conclusion of the work is provided in Section 6.

## 2. Materials and Methods

### 2.1. Materials

**Figure 1:**
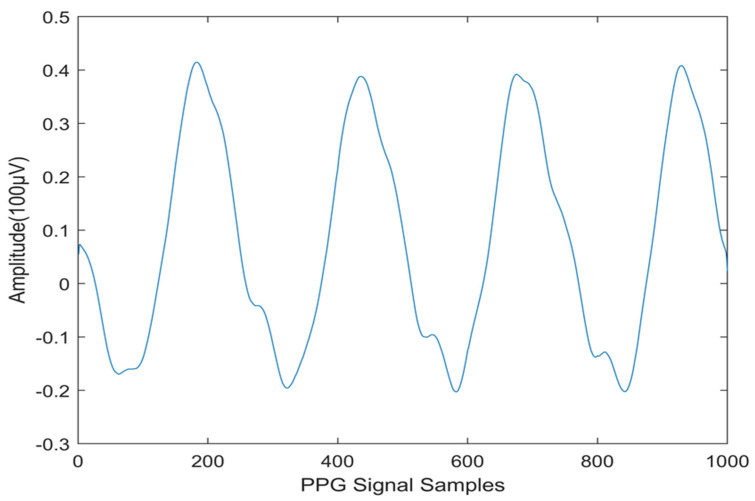
PPG signals for Normal Subject.

**Figure 2:**
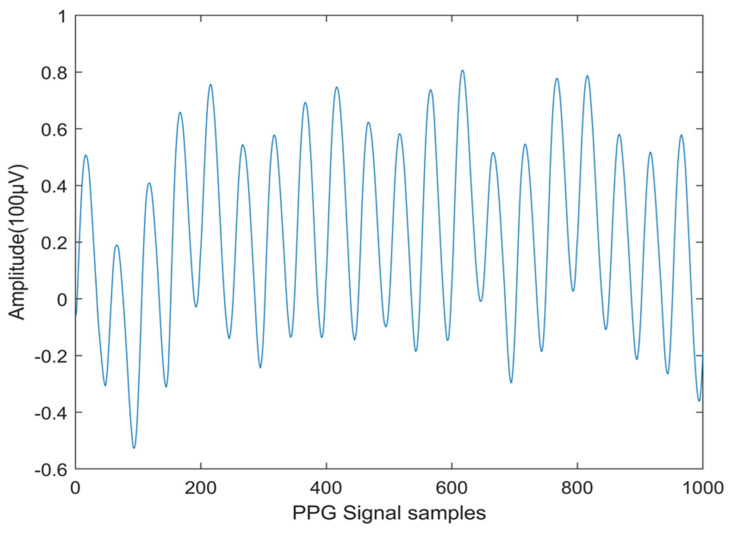
PPG signals for CVD Subject.

PPG data recordings with diverse wave shapes from the CapnoBase database have been used in this work. This database is a publicly available online resource that adheres to the IEEE TMBE pulse oximeter standard [30]. This article explores the CapnoBase dataset, utilizing the complete IEEE benchmark (41 records) for the experiment, with 20 records representing CVD and 21 representing normal conditions. The PPG signals are digitized at a rate of 200 samples per second. For analysis purposes, each one-second interval of the PPG signal is defined as a segment. Therefore, each patient has 720 individual segments for further examination. Consequently, each patient has 144,000 samples (720 segments × 200 samples per segment). The total number of CVD segments is [20 × 720 = 14,400], and in normal cases, the total number of segments is [21 × 720 = 15,120]. Therefore, in total, 29,520 one-second segments are available for analysis from the 41 cases. The PPG signals are analyzed based on signal segments across the patients. Beat-to-beat analysis is not included in this study. Noise components from the PPG signals were removed by utilizing Independent Component Analysis (ICA). This investigation is conducted using MATLAB R2020a software. Figure 1 shows a normal PPG signal, and Figure 2 depicts the PPG signal obtained from a CVD person.

### 2.2. Methods

CVD analysis using PPG signals is crucial due to the rising prevalence of cardiovascular diseases globally. PPG technology offers a non-invasive, cost-effective, and easily deployable method for continuous heart health monitoring, facilitating early detection and timely intervention. This can reduce the burden on healthcare systems and improve patient outcomes. The integration of PPG analysis with wearable devices and advanced machine learning algorithms enhances diagnostic accuracy and efficiency. As healthcare shifts towards personalized and preventive models, PPG-based CVD analysis becomes a pivotal tool in the fight against CVDs. Therefore, CVD detection from PPG signals is considered in this research work. The main objective of this work is to enhance CVD diagnosis through more precise classification systems. Effectively categorizing CVD data not only ensures patients receive appropriate care at reduced costs but also lowers their risk of developing the disease. Classifier accuracy tends to decrease when unimportant and noisy signals are present in recorded signals. To enhance the quality of the recorded PPG signals, an efficient filtering technique is implemented to eliminate unwanted noise and artifacts.

**Figure 3:**
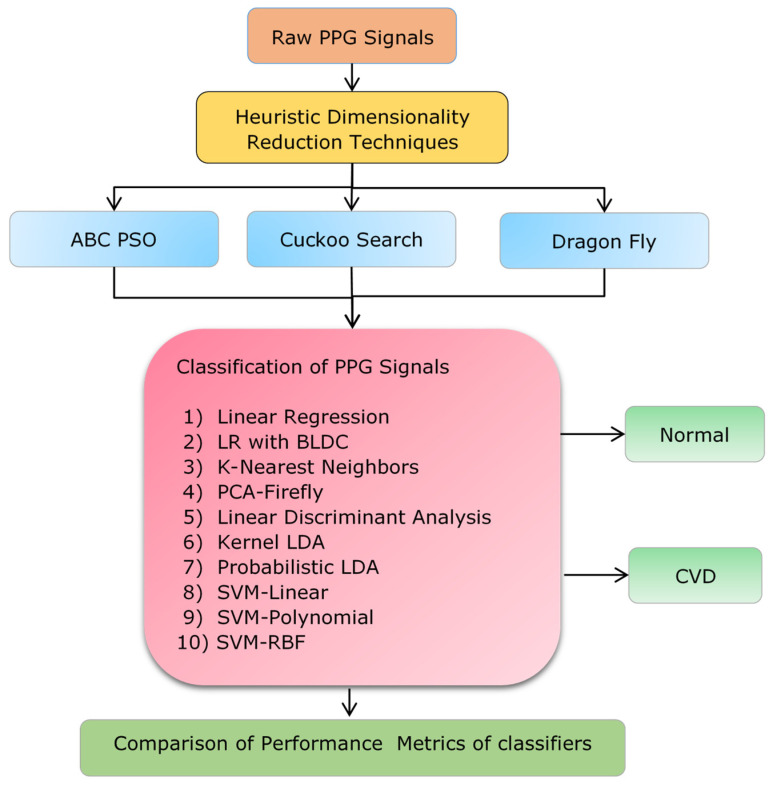
Detailed illustration of the workflow.

This research utilizes heuristic dimensionality reduction (DR) techniques as the initial step to reduce the dimensionality of the PPG data. Specifically, the research incorporates methods such as ABC-PSO (Artificial Bee Colony-Particle Swarm Optimization), the Cuckoo Search algorithm, and the Dragonfly algorithm. In the second stage, the optimized PPG data were input into ten different classifiers to detect cardiovascular disease from PPG signals. The classifiers’ performance is evaluated and differentiated using parameter metrics for heuristically optimized PPG values. Figure 3 provides a detailed illustration of the workflow.

## 3. Dimensionality Reduction Techniques

Now, each person has 144,000 samples of PPG signals (720 × 200). The objective of dimensionality reduction in PPG signals is to decrease the number of variables, thereby improving computational efficiency and reducing the risk of overfitting. High-dimensional data can be noisy and redundant, making it difficult for classifiers to accurately identify patterns associated with CVD. By extracting the most relevant features, dimensionality reduction techniques such as ABC-PSO, Cuckoo Search, and Dragonfly are employed to enhance the performance of machine learning models. 

### 3.1. ABC-PSO (Artificial Bee Colony-Particle Swarm Optimization)

**Figure 4:**
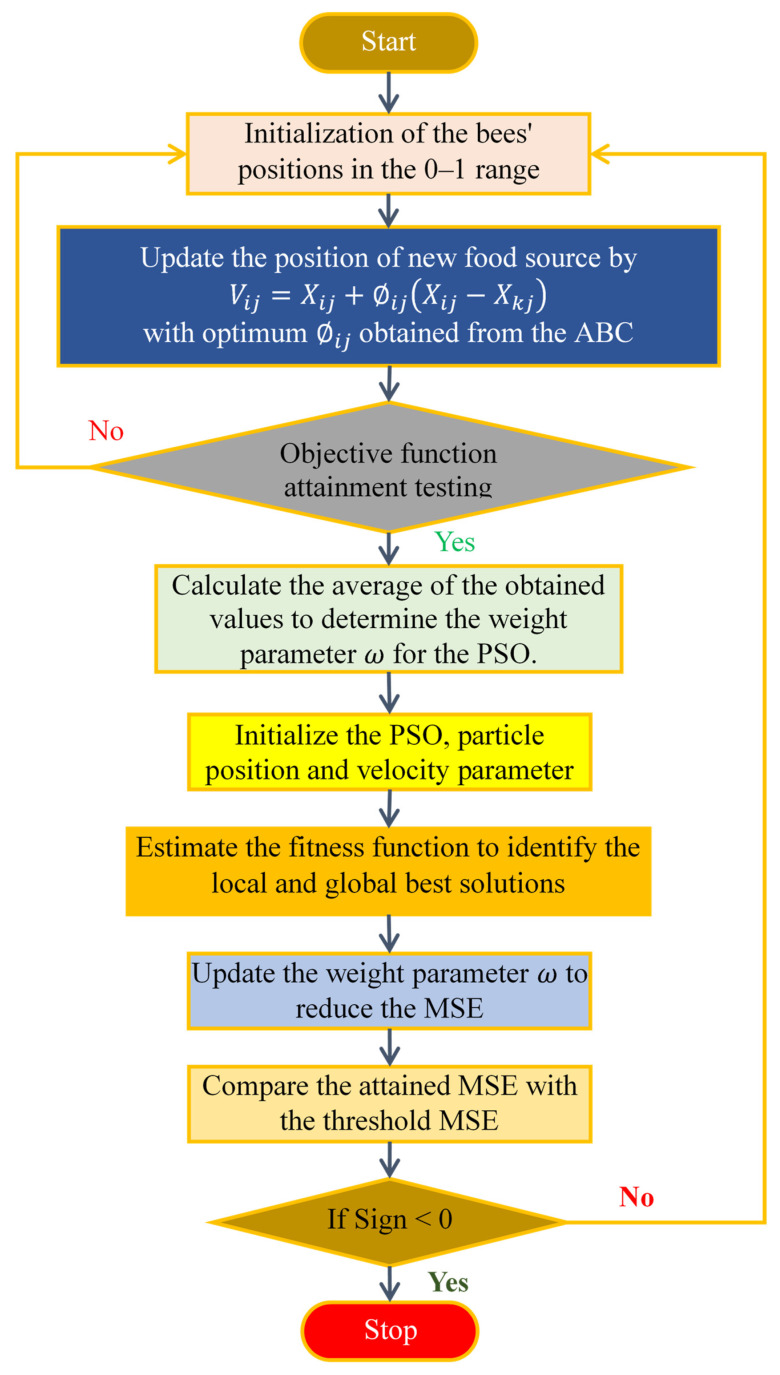
Flowchart of the ABC-PSO Algorithm.

ABC-PSO is a hybrid dimensionality reduction technique that combines the global search capabilities of the ABC algorithm with the local exploitation abilities of Particle Swarm Optimization (PSO). ABC emphasizes exploration through employed and onlooker bees, where solutions are iteratively improved. PSO, inspired by social behavior, optimizes by updating particle velocities based on personal best and global best solutions. By integrating these approaches, the hybrid algorithm aims to enhance exploration-exploitation balance, leveraging ABC’s local search capability and PSO’s global search efficiency. Figure 4 illustrates the flowchart of the ABC-PSO algorithm. This synergy enhances the efficiency of handling large datasets and improving the performance of data analysis [31].

ABC-PSO Algorithm Steps:

Initialization: Initialize bee and particle populations with random solutions. Set the number of employed bees, onlooker bees, and scout bees, as well as particle positions $x_{i}$ and velocities $v_{i}$.

Employed Bee Phase: Each employed bee explores new food sources (solutions) using the following:

(1) $$V_{ij}=X_{ij}+\emptyset_{ij}\left(X_{ij}-X_{kj}\right)$$

where $\emptyset_{ij}$ is a random number and $X_{kj}$ is a neighboring solution.

Onlooker Bee Phase: Onlooker bees in the algorithm choose their food sources probabilistically:

(2) $$P_{i\;}=\frac{f_{i}}{\sum_{i=1}^{N}f_{i}}$$

where $f_{i}$ is the fitness of solution $x_{i}$.

Scout Bee Phase: Abandon poor solutions and have scout bees search for new random solutions.

PSO Update: Update particle velocities and positions using the following equations [32]:

(3) $$v_{i}\left(t+1\right)=\omega v_{i}\left(t\right)+c_{1}r_{1}\left(p_{i}-x_{i}\right)+c_{2}r_{2}\left(g-x_{i}\right)$$

(4) $$x_{i}\left(t+1\right)=x_{i}\left(t\right)+v_{i}\left(t+1\right)$$

where $p_{i}$ is the personal best position, g is the global best position, ω is the inertia weight, $r_{1}$ and $r_{2}$ are random numbers, $c_{1}$ and $c_{2}$ are acceleration coefficients.

Evaluation and Selection: Evaluate new solutions and select the best ones based on fitness.

Convergence Check: Repeat the steps until stopping criteria, such as maximum iterations or a convergence threshold are met.

### 3.2. Cuckoo Search Algorithm (CSA)

**Figure 5:**
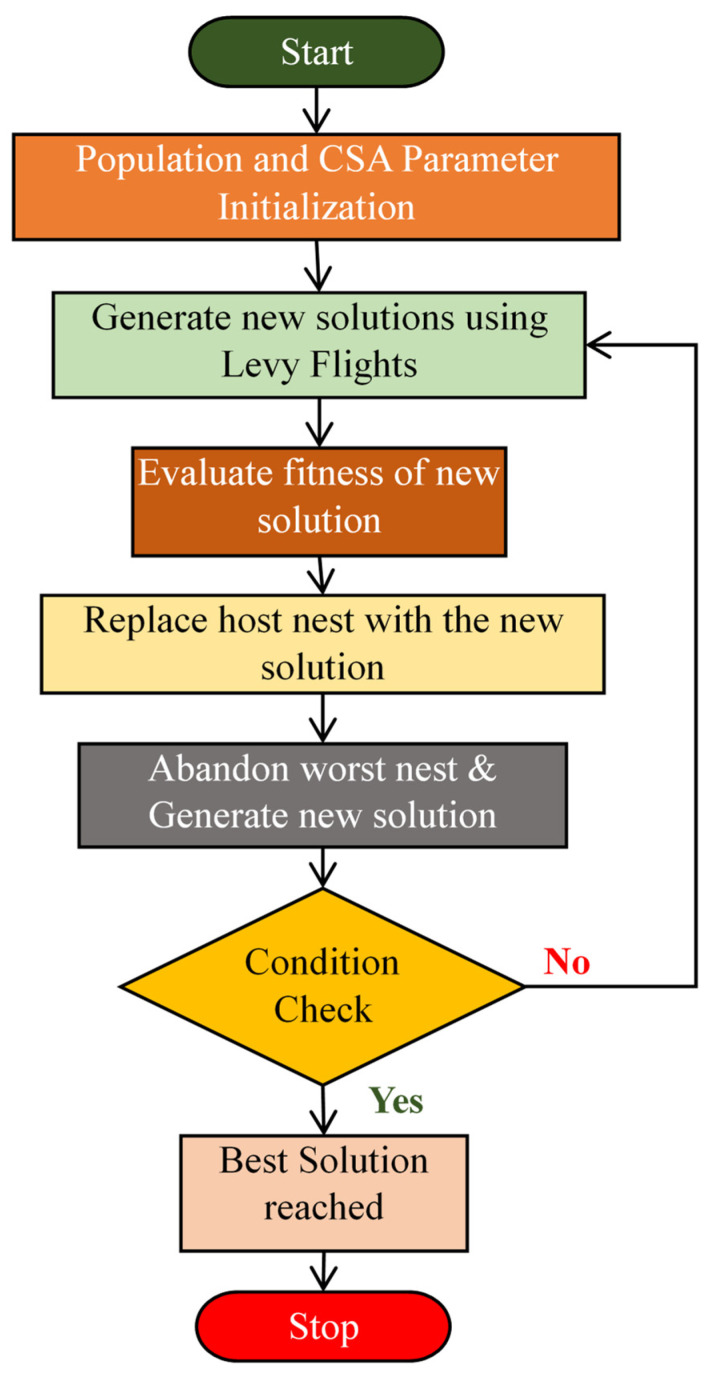
Flowchart of the Cuckoo Search Algorithm.

It is a nature-inspired metaheuristic optimization technique established by Xin-She Yang and Suash Deb in 2009 [33]. Figure 5 illustrates the methodology of the CSA. It is effective for dimensionality reduction by finding optimal feature subsets. The feature subset is chosen that optimizes a performance metric, specifically aiming for the lowest MSE. This algorithm draws inspiration from the reproductive strategy of cuckoo birds. It simulates the unique behavior of cuckoos, which lay their eggs in the nests of other bird species. The algorithm employs both randomization and local search techniques to guide the cuckoos towards the optimal solution. A notable characteristic of CSA is its utilization of Levy flights, which are random walks characterized by a heavy-tailed distribution. This method regulates the movement of cuckoos, enabling efficient exploration of the feature space while swiftly navigating towards promising regions. Therefore, CSA is selected as a DR technique for its capability to balance exploration and exploitation and effectively manage high-dimensional data. The CSA commences its process by generating an initial population of cuckoos with random positions in the feature space. The position of each cuckoo in the population is then updated using the following equation [34]:

(5) $$z_{k}^{new}=z_{k}^{old}+\alpha *Levy(\lambda )$$

where the cuckoo’s previous position is represented by $z_{k}^{old}$, while its updated position is denoted as $z_{k}^{new}$, where *α* is a scaling factor, and Levy(λ) represents a step drawn from a Levy distribution.

The cuckoos’ random search patterns are modeled by a concept called the Lévy flight, described by the following equation:

(6) s=τ∗r−1μ

Here, ‘s’ represents the step size, τ is a parameter that dictates the scale of the Levy flight, and shape of the Levy flight is determined by the parameter μ. A random number ‘r’ chosen uniformly between 0 and 1. The optimal nest in the population is chosen based on the fitness value. MSE is used to derive the fitness value, and the best nest serves as the starting point for the next iteration of the algorithm. Also, abandon a fraction ${\text{‘}P}_{a}\text{’}$ of the worst nests and generate new solutions to replace them. This process helps keep the search space varied, allowing the algorithm to explore new potential solutions. By using ${\text{‘}P}_{a}\text{’}$ to periodically introduce new random solutions, the algorithm maintains a balance between exploration and exploitation, which is crucial for effective dimensionality reduction and optimization.

### 3.3. Dragonfly Algorithm 

**Figure 6:**
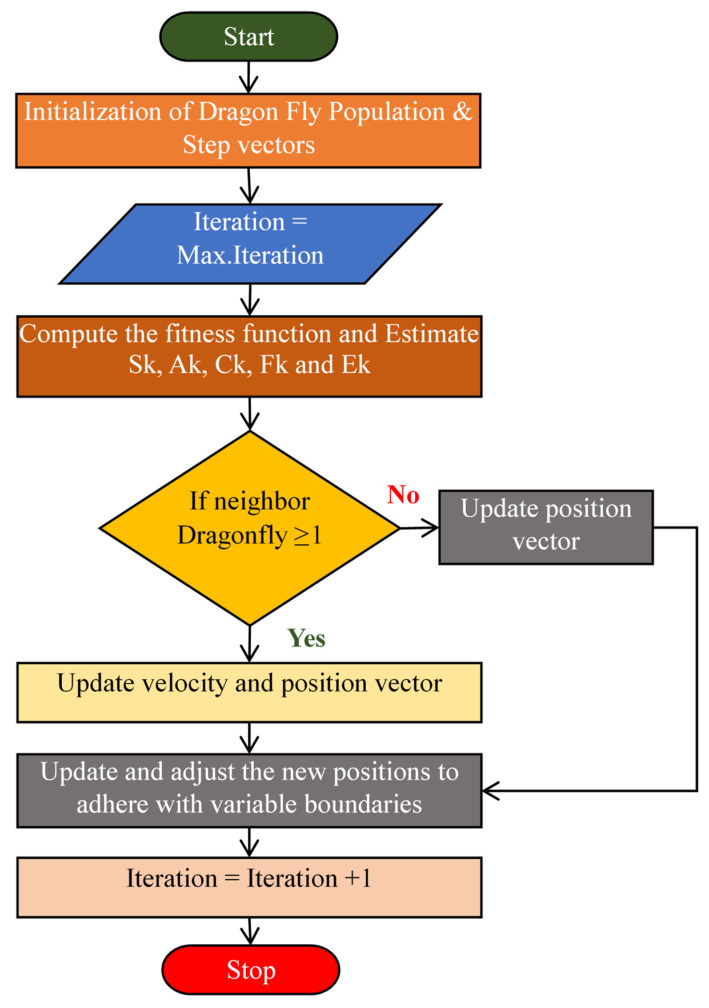
Flow Chart representation of the Dragonfly Algorithm.

The Dragonfly Algorithm (DFA) is a heuristic optimization technique inspired by the fascinating static and dynamic swarming behaviors of dragonflies. DFA was developed by Seyedali Mirjalili in 2015 [35], and this algorithm mimics the way dragonflies search for food and avoid predators. Figure 6 presents the DFA flowchart. Dragonflies display two main behaviors: static, where they gather in small groups and hover around a target, and dynamic, where they collectively move towards a distant target. These behaviors are translated into mathematical models that help in exploring and exploiting the search space efficiently. It is effective for dimensionality reduction by finding optimal feature subsets through exploration and exploitation of the search space. As represented by Rahman et al. [36], separation, alignment, cohesion, attraction to food, and distraction from the enemy are important features of the DFA. In the following equations, q represents the current position of an individual dragonfly, $q_{j}$ denotes the position of the $j^{th}$ dragonfly, and ‘M’ indicates the total number of neighboring dragonflies.

Separation: It prevents dragonflies from crowding together by maintaining a minimum distance from each other.

(7) $$S_{k}=-\sum_{j=1}^{M}q-q_{j}$$

The symbol $S_{k}$ represents the motion of separation exhibited by the $k^{th}$ individual.

Alignment: It refers to the tendency of dragonflies to match their velocity with that of their neighbors.

(8) $$A_{k}=\frac{\sum_{j=1}^{M}V_{j}}{M}$$

where $A_{k}$ represents the alignment motion for the $k^{th}$ individual, $V_{j}$ indicates $j^{th}$ neighboring individual dragon velocity.

Cohesion: is the behavior that drives dragonflies to move towards the center of their neighboring individuals.

(9) $$C_{k}=\frac{\sum_{j=1}^{M}q_{j}}{M}-q\;$$

Attraction: It is the tendency of dragonflies to move towards food sources

(10) $$F_{k}=q^{+}-q$$

where $F_{k}$ represents the attraction of nutrition source for the $k^{th}$ individual flyand the position of the food source is denoted as $q^{+}$.

Distraction: It is the tendency of dragonflies to move away from enemies.

(11) $$E_{k}=q^{-}+q$$

where $E_{k}$ represents the distraction motion caused by the enemy for the $k^{th}$ individual and the location of the enemy is denoted as $q^{-}$.

The positions of artificial dragonflies inside the designated search area are revised using the current position vector (q) and the step vector (∆q). The direction of their movement is determined by the step vector (∆q ), and it is calculated as,

(12) $$∆q_{t+1}=\left(sS_{k}+aA_{k}+cC_{k}+fF_{k}+eE_{k}\right)+\omega q_{t\;}$$

where iteration number is denoted as ‘t’, ‘ω’ denotes the inertia weight and weights assigned to separation, alignment, cohesion, attraction, and enemy, are denoted as  s,  a,  c, f, and  e respectively. The exploitation and exploration phases can be achieved by modifying the weights, once the step vector calculation is completed, calculation of the position vectors commences as below:

(13) $$q_{t+1}=q_{t}+∆q_{t+1}$$

Table 2 demonstrates the selection of the optimal parameters for heuristic algorithms.

Statistical metrics such as Pearson Correlation Coefficient (PCC), Sample Entropy, and Canonical Correlation Analysis (CCA), mean, variance, kurtosis, and skewness were employed to identify distinct characteristics in the PPG signals between the different classes, allowing for faster analysis as presented in Table 3.

It is perceived from Table 3 that the calculated mean values are notably lower for both normal and CVD cases under ABC PSO DR technique and also for cuckoo search normal category. For cuckoo search CVD class, a higher mean value is attained. Under dragon fly optimization, negative mean values are achieved for both Normal and CVD. Skewness and kurtosis values are considerably skewed for both normal and CVD for all three DR techniques. Table 3 indicates that the sample entropy values are same across classes with the exception of the cuckoo search DR technique in the CVD case. Table 3 further illustrates that the low PCC values suggest that the optimized features exhibit nonlinearity and lack correlation across different classes. If CCA values exceed 0.5, significant correlation between the classes can be expected. Table 3 shows that the dragon fly optimization is highly related to all of the classes compare to other DR techniques. It also shows that the ABC PSO DR technique has a lower correlation with the other classes.

**Figure 7:**
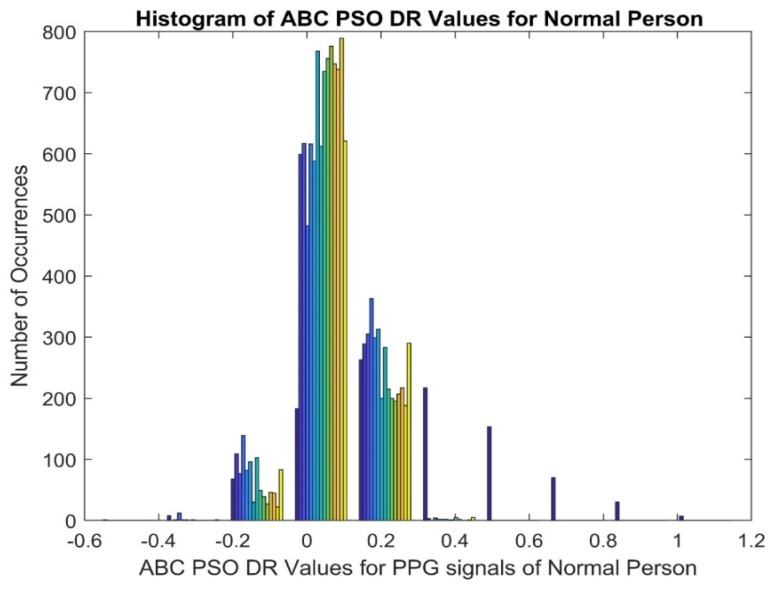
Histogram of ABC PSO DR PPG signals for Normal Person.

**Figure 8:**
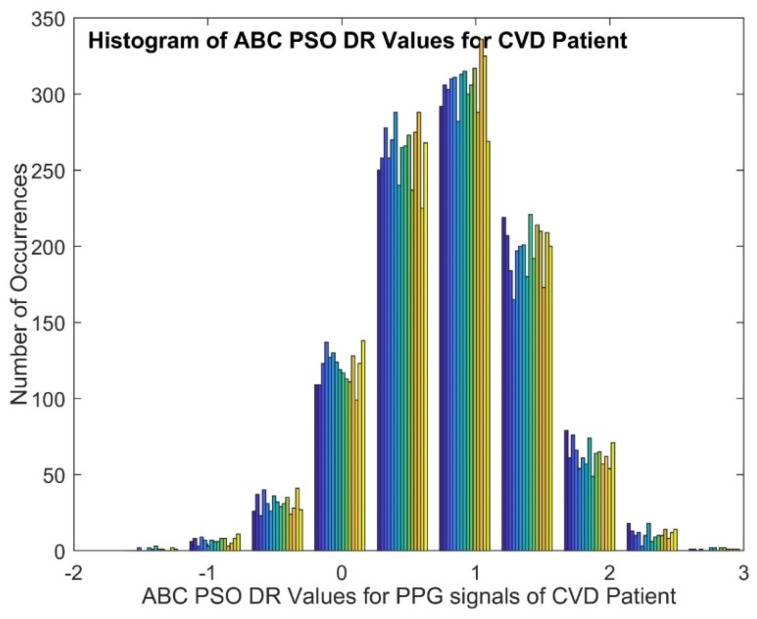
Histogram of ABC PSO DR PPG signals for CVD Patient.

**Figure 9:**
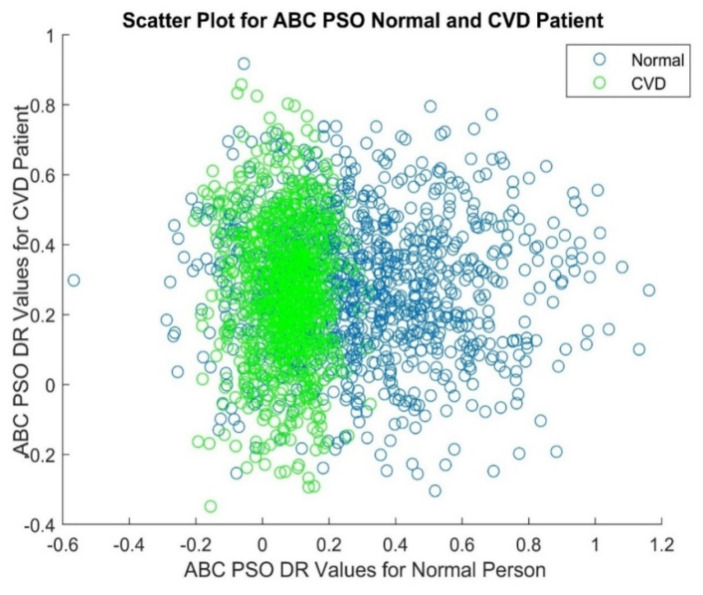
Scatter Plot of ABC PSO based DR values of PPG signals.

**Figure 10:**
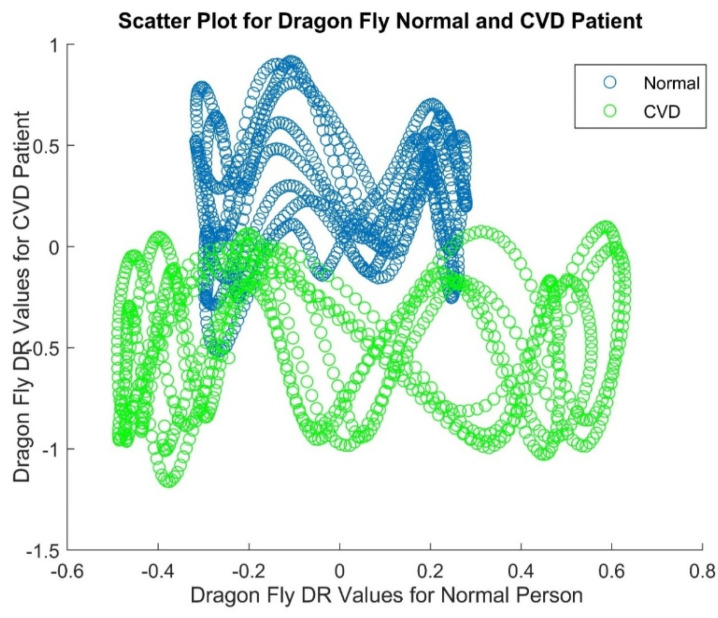
Scatter Plot of Dragon Fly based DR values of PPG signals.

Figure 7 depicts the histogram plot of ABC PSO DR values for a normal person and it gives that the histogram with higher peaks in the middle and lower at either end. Figure 8 displays the histogram plot of ABC PSO DR values for a CVD patient, indicating that the histogram has a normal and Gaussian distribution. The scatter plot of ABC PSO DR values of normal and CVD data is depicted in Figure 9, which clearly indicates that the DR features are highly merged among the classes at the center and lesser overlapping at the end.The scatter plot of Dragon Fly DR values is portrayed in Figure 10. It shows that the lesser overlapping is present among the normal and CVD classes.

## 4. Classifiers for Classification of CVD from Dimensionality Reduced Values

### 4.1. Linear Regression as a Classifier

Linear regression (LR) falls under the category of supervised learning techniques. It is primarily employed to forecast continuous numerical outcomes. This algorithm establishes a linear relationship between input features and the target variable, allowing it to make predictions on new, unseen data based on the patterns learned from the training set. The linear equation is defined by a set of coefficients that are estimated using the training data. Linear regression is is primarily designed for predicting continuous numerical outcomes, it demonstrates versatility in its application. Through appropriate modifications, this algorithm can be effectively repurposed to address classification problems. The basic linear regression model is defined as follows [37]:

(14) $$q=b_{0}+b_{1}x_{1}+b_{2}x_{2}+\ldots +b_{n}x_{n}+\epsilon$$

where $x_{1}$, $x_{2}$, …, $x_{n}$ are independent variables, q is the dependent variable, $b_{0}$ is the intercept, the coefficients are $b_{1}$, $b_{2}$, …, $b_{n}$, and the error term is ϵ. For classification, the predicted y values can be thresholded to determine class membership. In this research, we set a threshold of 0.5 to classify PPG data as either normal or CVD. Predictions greater than 0.5 are classified as CVD, while those less than 0.5 are considered normal. This approach is simple but can be limited by linear regression’s assumptions and the nature of classification problems.

### 4.2. Linear Regression with BLDC

LR and LR-BLDC models use the same linear relationship. However, LR is used for regression tasks with continuous outputs, whereas LR-BLDC adapts this relationship for binary classification by applying a threshold to the predicted values. The Bayesian Linear Discriminant Classifier operates as a probabilistic generative model specifically tailored for classification challenges. It involves estimating the class-conditional probability distribution of the input variables for each class and using Bayes’ rule to calculate the posterior Probability of each class based on the input variables [38]. To combine linear regression and BLDC, the predicted output of the LR model is used as an input to the BLDC model. The linear regression classifier output is used to estimate the mean of the class-conditional probability distribution for each class in the BLDC model.

### 4.3. K-Nearest Neighbor as a Classifier

KNN (K-Nearest Neighbors) is a machine learning algorithm that classifies data based on the nearest neighbors. The parameter “K” represents the number of neighbors considered. The core idea is to classify a data point by finding the closest training points to it, based on a similarity measure [39]. After identifying the K number of nearest neighbors, it allots the input point to the class with the maximum frequency among its K-nearest neighbors. It does not need a different phase for training. Instead, the algorithm retains the complete training dataset and employs it to classify new data points. Weighted KNN is a classification algorithm that assigns weights to the neighbors based on their distance to the query point. In the KNN algorithm, for a given point z, the algorithm finds the *K* data points closest to a new point by measuring their distance using a metric like Euclidean distance [40]:

(15) $$d\left(z,z_{i}\right)=\sqrt{\sum_{j=1}^{m}{\left(z_{j}-z_{ij}\right)}^{2}}$$

**Figure 11:**
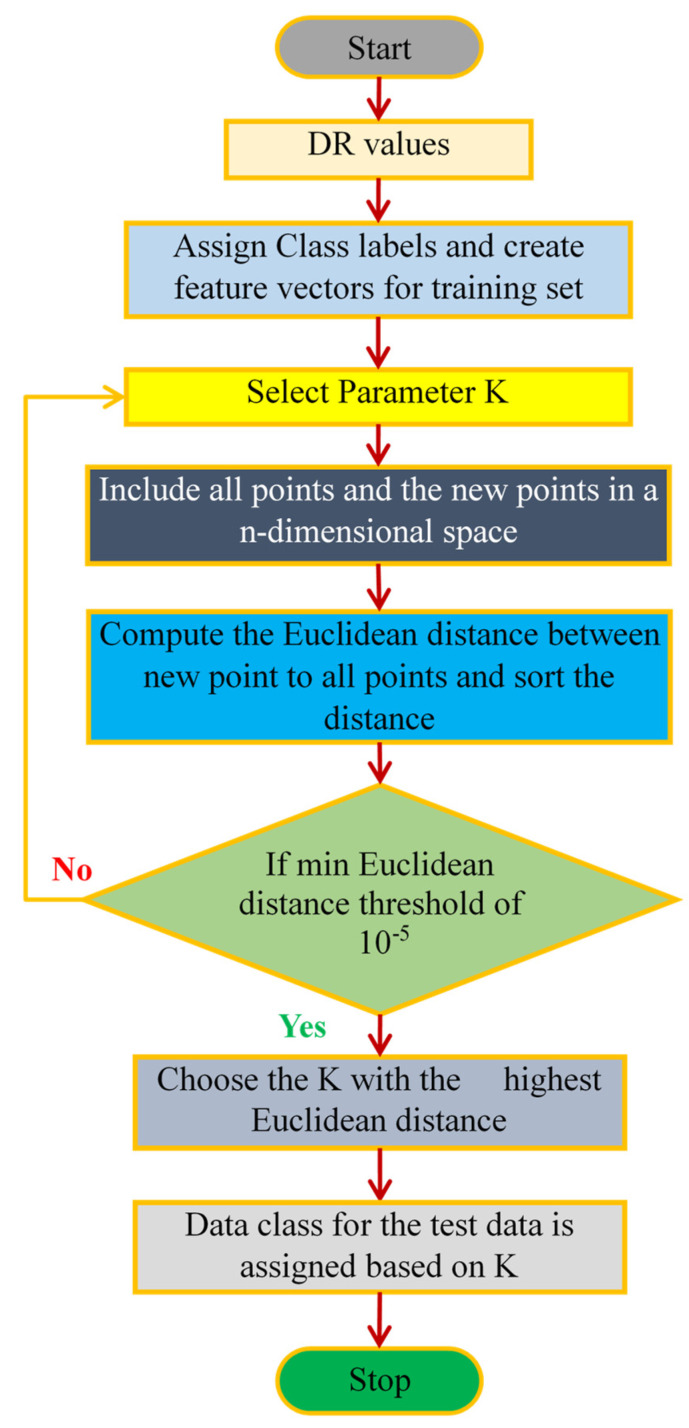
Flow diagram of the KNN algorithm.

In weighted KNN, each neighbor $z_{i}$ is assigned a weight $w_{i}$ inversely proportional to its distance from z:

(16) $$w_{i}=\frac{1}{d\left(z,z_{i}\right)+\varepsilon }$$

where ε is a small constant to avoid division by zero. The algorithm predicts the class of the query point by considering the most frequent class among its K closest neighbors:

(17) $$\hat{b}=\arg{max}_{c}\sum_{i\in M_{K\left(z\right)}}w_{i}.I(b_{i}=c)$$

where $M_{K\left(z\right)}$ denotes the set of K nearest neighbors, the class label of neighbor $z_{i}$ is $b_{i}$ and the indicator function is I (⋅). This method improves classification accuracy by giving more influence to closer neighbors. Figure 11 depicts the flow diagram of the KNN algorithm.

### 4.4. PCA-Firefly

The hybrid PCA firefly technique is used to choose the most relevant features while removing irrelevant ones, thereby optimizing accuracy to its fullest extent. The PPG dataset undergoes dimensionality reduction using the PCA algorithm, which effectively reduces the quantity of stochastic variables to a concise group of primary variables. This approach greatly enhances the accuracy of the prediction results [41]. To further optimize the process, the firefly optimization algorithm is utilized to select the most appropriate attributes from this refined reduced dataset.

PCA simplifies data by finding a new set of features that are not related to each other by solving the following eigenvalue problem:

(18) $$Y^{T}Y_{v}=\lambda v$$

where Y is the data matrix, v  are the eigenvectors (principal components), and λ are the eigenvalues.

**Figure 12:**
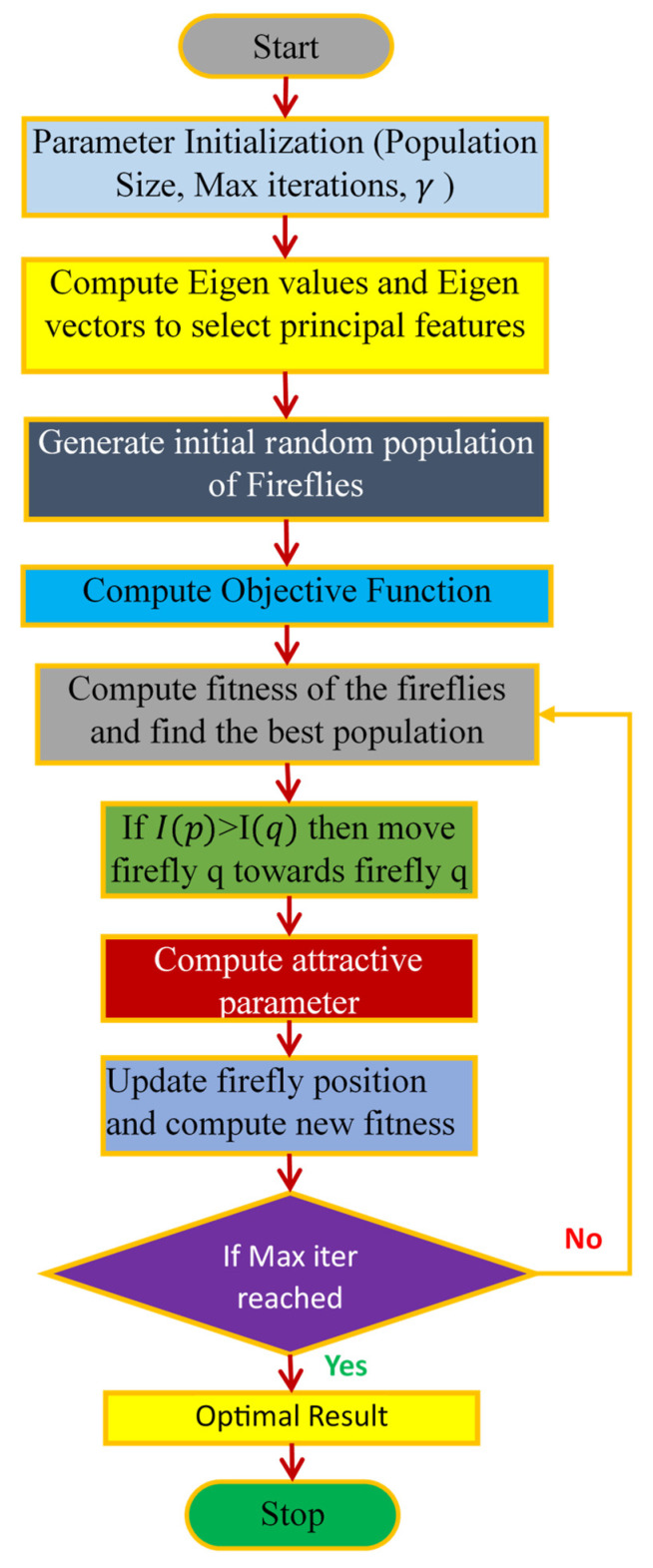
Workflow of the PCA-Firefly algorithm.

The Firefly Algorithm is a clever problem-solving method that mimics how fireflies communicate with their flashes, optimizes the selection of principal components. Each firefly represents a potential solution with an intensity I proportional to its fitness. The attractiveness α between fireflies i and j is given by the following [42]:

(19) $$\alpha_{ij}=\alpha_{0}e^{-\gamma p_{ij}^{2}}$$

where $\alpha_{0}$ is the attractiveness at p = 0, γ is the light absorption coefficient, and $p_{ij}$ is the distance between fireflies i and j. Fireflies are drawn to brighter ones, constantly adjusting their location:

(20) $$x_{i}=x_{i}+\alpha_{ij}\left(x_{j}-x_{i}\right)+\beta \epsilon_{i}$$

where β is a randomization parameter, and $\epsilon_{i}$ is a random vector. This process iteratively refines the feature selection, enhancing classification performance. Figure 12 presents the workflow of the PCA-Firefly algorithm.

### 4.5. Linear Discriminant Analysis as a Classifier

Linear Discriminant Analysis (LDA) is a dimensionality reduction technique used for classification, aiming to find the best way to combine features to distinguish between different groups. LDA assumes that each class follows a Gaussian distribution with a shared covariance matrix. The steps of LDA are as follows [43]:

1. Calculate the mean vector for each class

(21) $$\mu_{z}=\frac{1}{M_{z}}\sum_{i\in C_{z}}x_{i}$$

2. Calculate the within-class scatter matrix

(22) $$S_{w}=\sum_{z=1}^{M}\sum_{i\in C_{z}}\left(x_{i}-\mu_{z}\right){\left(x_{i}-\mu_{z}\right)}^{T}$$

3. Calculate the between-class scatter matrix

(23) $$S_{b}=\sum_{z=1}^{M}M_{z}\left(\mu_{z}-\mu \right){\left(\mu_{z}-\mu \right)}^{T}$$

where μ is the overall mean of the dataset.

4. Solve the generalized eigenvalue problem

(24) $$S_{w}^{-1}S_{b}v=\lambda v$$

The eigenvectors v corresponding to the largest eigenvalues λ form the transformation matrix. Project the data onto this lower-dimensional space to maximize class separability. In classification, the new data point x is projected and assigned to the class with the closest mean in this reduced space.

### 4.6. Kernel LDA as a Classifier

Kernel Linear Discriminant Analysis (KLDA) is an extension of LDA that incorporates the kernel trick to handle nonlinear data. The objective of this technique is to identify an optimal projection of the data in a high-dimensional feature space, which is created through the application of a kernel function. This projection is designed to simultaneously maximize the separation between different classes (between-class scatter) and minimize the spread within each individual class (within-class scatter).

The objective function of KLDA is formulated as follows [44]:

(25) $${max}_{v}\frac{v^{T}S_{b}v}{v^{T}S_{w}v}$$

where $S_{b}$ is the between-class scatter matrix and $S_{w}$ is the within-class scatter matrix in the kernel space. The solution v corresponds to the eigenvector associated with the largest eigenvalue of the generalized eigenvalue problem $S_{w}v={\lambda S}_{b}v$. KLDA is effective for nonlinear data leveraging a kernel function to implicitly project the original input space into a higher-dimensional feature space, where data separation is potentially easier to achieve. It has found applications in various fields where nonlinear relationships among data features are prevalent.

### 4.7. Probabilistic LDA as a Classifier

Probabilistic Linear Discriminant Analysis (ProbLDA) is an extension of Linear Discriminant Analysis (LDA) that models class distributions probabilistically. ProbLDA assumes that each class follows a Gaussian distribution and utilizes Bayesian principles for classification [45]. The class mean, within-class scatter matrix and between-class scatter matrix are computed as per the Equations (21)–(23). Then, for each class ‘z’ Gaussian distributions can be assumed as follows:

(26) pxCz=12πd2Ʃ12exp⁡−12x−μzTƩ−1x−μz

where Ʃ is the Shared covariance matrix estimated as $S_{w}$.

To calculate the posterior probabilities for classification, Bayes’ theorem can be used as per the below equation:

(27) $$p\left(C_{z}|x\right)=\frac{p\left(x|C_{z}\right)\;p\left(C_{z}\right)}{p(x)}$$

Here $p(C_{z}$) prior probability of class z and p(x) is the marginal probability of x. Assign the new data point x to the class with the highest posterior probability.

(28) $$\hat{g}=arg\;{max}_{z}\;p\left(C_{z}|x\right)$$

ProbLDA integrates the strengths of LDA with probabilistic modeling, providing a robust framework for classification.

### 4.8. Support Vector Machine as a Classifier

The Support Vector Machine (SVM) is a sophisticated supervised learning technique primarily employed for classification purposes.

SVM treats the input data as an n-dimensional feature vector space and seeks to find an (n − 1) dimensional hyperplane that divides the space into two regions. This hyperplane is positioned to maximize the minimum distance between any data point and the boundary [46].The n-dimensional input data $x_{j\;}$(where j = 1, 2, ….., N, with N representing the number of samples) is assigned labels $y_{j}=1\;$ for class 1 and $y_{j}=-1\;$ for class 2 using the $y_{j}$ matrix. For linearly separable data, a hyperplane can be defined as $f\left(x\right)=0$ [47].

(29) $$f\left(x\right)=w.x+d=\sum_{j=1}^{n}w_{j}x_{j}+d=0$$

w is an  n-dimensional vector, and d is a scalar. These parameters define the position of the hyperplane, establishing clear boundaries for classification. In SVM, input data are mapped to a higher-dimensional feature space using kernel functions to address multiclass classification problems. SVM aims to achieve high generalization by effectively separating classes based on the training data.

In this research, SVM with three different kernels are used: Radial Basis Function (RBF), linear, and polynomial [48].

SVM-Linear:

(30) $$\ker\left(z_{i},z_{j}\right)={z_{i}}^{T}z_{j}$$

SVM-Polynomial:

(31) $$\mathrm{Ker}\left(z_{i},z_{j}\right)={\left(1+{ϒz}_{i}^{T}.z_{j}\right)}^{q},\;ϒ>0$$

where the degree of polynomial kernel is denoted as *q* and ‘*ϒ*’ denotes the gamma term in the kernel function.

SVM-RBF:

(32) $$\ker\left(z_{i},z_{j}\right)=\exp\left(-ϒ{\left\|z_{i}-z_{j}\right\|}^{2}\right),ϒ>0$$

where, $\left\|z_{i}-z_{j}\right\|$ is the Euclidean distance between two input vectors ${\;z}_{i}$ and ${\;z}_{j}$.

These kernel functions operate by projecting the input data into a space of increased dimensionality. This transformation facilitates the identification of an optimal hyperplane that effectively delineates between different classes.

## 5. Results and Discussion

### 5.1. Training and Testing of the Classifiers

This research work employed a data partitioning strategy wherein 90% of the available dataset was dedicated to the model’s training process, while the remaining 10% was set aside for subsequent testing and validation. To assess the classifier’s efficacy and reliability, we implemented a ten-fold cross-validation approach. A True Positive (TP) occurs when the classifier correctly identifies a positive sample, while a True Negative (TN) is when it accurately labels a negative sample. A False Positive (FP) occurs when the model erroneously classifies a negative instance as positive, whereas a False Negative (FN) happens when the model incorrectly identifies a positive instance as negative. The Mean Squared Error (MSE) is defined by the following mathematical formula:

(33) $$MSE=\frac{1}{M}\sum_{k=1}^{M}{\left(O_{k}-T_{i}\right)}^{2}$$

where M denotes the complete set of data points within the PPG dataset, and it is assumed as 1000. The target value of model ‘i’ is designated by $T_{i}$, where the range of ‘i’ varies from 1 to 15; at a specific time, $O_{k}$ represents the observed value. The training was conducted in a manner that significantly reduced the classifier’s Mean square error to a minimal value.

Table 4 presents the testing and training MSE for classifiers using three different DR techniques. The training MSE ranges from 10^−5^ to 10^−10^, in contrast, the testing phase yielded MSE values ranging from 10^−3^ to 10^−9^. The SVM-RBF classifier under the ABC-PSO dimensionality reduction method achieved the lowest training of 1.92 × 10^−10^ and testing MSE value of 2.45 × 10^−9^. The DFA DR method yields somewhat reduced MSE values for both training and testing phases across the classifiers, when compared to the alternative DR approaches evaluated in this work. Similarly, for Cuckoo Search DR features the LR classifier yields the minimal testing MSE value of 1.37 × 10^−8^, due the correct labeling of PPG signal for both CVD and normal subjects. The PCA Firefly classifier is plugged with a high number of FP and FN cases, resulting in the highest testing MSE of 6.08 × 10^−3^. Similarly, for Dragon Fly DR values, the SVM-RBF classifier achieves the minimum testing Mean square error of 3.62 × 10^−9^. This superior performance is attributed to its accurate labeling of PPG signals for both CVD and normal subjects. Conversely, the SVM-Linear classifier shows poorer performance, resulting in a much higher testing MSE of 9.03 × 10^−3^. This higher error rate is due to the SVM-Linear misclassifying a significant number of cases, producing both false negatives and false positives.

**Figure 13:**
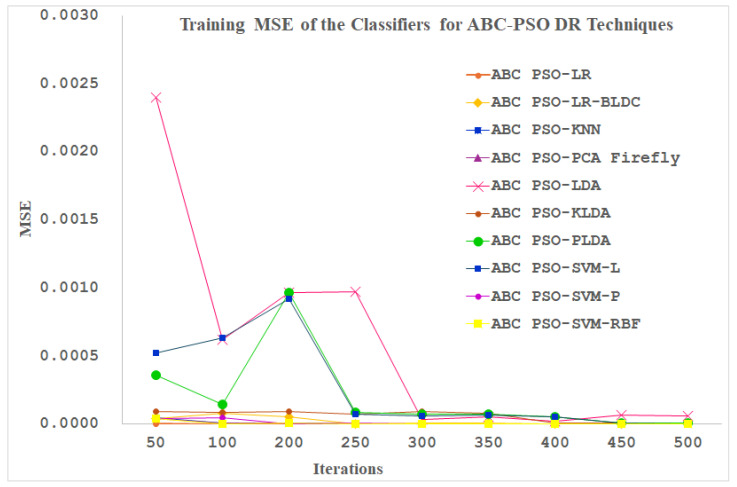
Training MSE of the Classifiers for ABC-PSO DR Techniques.

Figure 13 displays the training performance of the ten classifiers using the ABC-PSO dimensionally reduced PPG signals as input. As shown in Figure 13, the maximum number of iterations is set to 500. The Logistic Regression, SVM-Polynomial and SVM- RBF Classifiers reached the minimum MSE value at 50 iterations itself. All the other classifiers are settled at minimum MSE values after 250 iterations. The LDA, SVM-linear and PLDA classifiers depict a distinctive performance apart from all the other classifiers up to 350 iterations.

**Figure 14:**
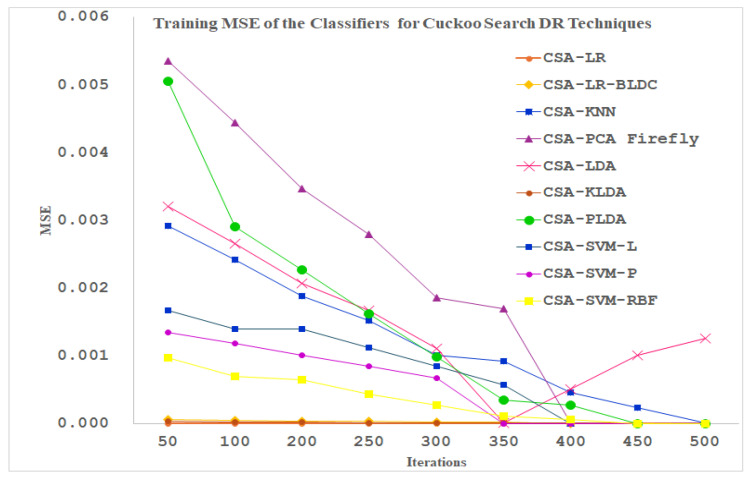
Training MSE of the Classifiers for Cuckoo Search DR Techniques.

Figure 14 displays the training performance of the ten classifiers for the cuckoo search dimensionally reduced PPG signals as the input. As exhibited in Figure 14, the maximum number of iterations is fixed at 500. The Linear Regression, LR-BLDC and KLDA Classifiers reached the minimum MSE value at 50 iterations itself. All the other classifiers are settled at minimum MSE values after 300 iterations. The LDA, PLDA and PCA-Firefly classifiers depict a peculiar performance apart from all the other classifiers up to 350 iterations.

**Figure 15:**
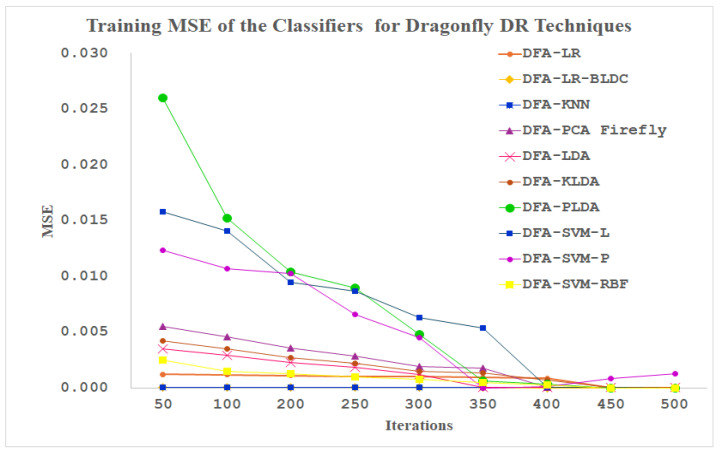
Training MSE of the Classifiers for Dragonfly DR Techniques.

Figure 15 explores the training MSE performance of the ten classifiers for the dragonfly dimensionally reduced PPG signals as the input. As mentioned in Figure 15, the maximum number of iterations is fixed at 500. The LR-BLDC and SVM-Linear classifiers reached the minimum MSE value at 50 iterations itself. All the other classifiers are settled at minimum MSE values after 300 iterations. The PLDA, SVM-linear and SVM-Polynomial classifiers are depicting a unique performance apart from all the other classifiers up to 350 iterations.

### 5.2. Optimal Parameters Selection for Classifiers

When selecting the target values for the binary classification of PPG dataset (CVD and Normal class), a deliberate choice is made to assign the target ($T_{CVD})\;$ values towards the upper end of the 0 to 1 range. The criteria used to select $T_{CVD}$ is as follows:

(34) $$\frac{1}{Y}\sum_{p=1}^{Y}\mu_{p}\leq T_{CVD}$$

The complete set of CVD PPG data features, denoted as (Y), underwent a normalization process with mean $\mu_{p}$. For the target $T_{Normal}$ values, a deliberate choice is made to assign them towards the lower end of the 0 to 1 range. The selection criteria for determining the value of $T_{Normal}$ are governed by the following parameters:

(35) $$\frac{1}{X}\sum_{q=1}^{X}\mu_{q}\leq T_{Normal}$$

The complete set of normal PPG data features, denoted as (X), underwent a normalization process with mean $\mu_{p}$. For optimal categorization, the following equation is used:

(36) $$\left\|T_{CVD}-T_{Normal}\geq 0.5\right\|$$

Based on the condition provided in (36), in this research work, the targets have been set at 0.1 for normal and 0.85 for CVD. Table 5 details the iterative process of selecting optimal parameters for the classifier during its training phase. A maximum of 1000 iterations is allowed to control the convergence criteria.

### 5.3. Performance Analysis of the Classifier

The classifiers’ effectiveness is assessed using a comprehensive set of metrics, such as Accuracy, Good Detection Rate (GDR), F1 Score, Kappa, Matthews Correlation Coefficient (MCC), Error rate, and Jaccard Index. The following formula [49,50] used for evaluating the overall effectiveness of the classification method.

(37) $$Accuracy=\frac{TN+TP}{TP+TN+FP+FN}\times 100\%$$

(38) $$Good\;detection\;rate\;(GDR)=\frac{\left(TN+TP\right)-FN}{(TN+TP)+FP}\times 100\%$$

(39) $$Error\;Rate=\frac{FP+FN}{TP+TN+FP+FN}\times 100\%$$

(40) $$Kappa=\frac{\left(\frac{TP+TN}{100}-E_{acc}\right)}{\left(1-E_{acc}\right)}$$

where, $E_{acc}=(((TP+FP)/100)*(TP+FN)/100+(((TN+FP)/100)*((TN+FN)/100)$

(41) $$MCC=\frac{\left(TN.TP\right)-(FN.FP)}{\sqrt{\left(TN+FN\right).\left(TN+FP\right)\left(TP+FN\right).\left(TP+FP\right)}}\times 100\%$$

(42) $$F1\;Score=\frac{2TP}{2TP+FP+FN}\times 100\%$$

(43) $$Jaccard\;Index=\frac{TP}{TP+FP+FN}\times 100\%$$

Table 6 presents the results of the performance analysis for classifiers with three dimensionality reduction techniques. The results in Table 6 reveal that the classifier SVM-RBF for ABC PSO dimensionality reduction technique achieved very high scores on all benchmark metrics, including a superior accuracy of 95.12%, a F1 score and GDR of 95%, and a lowest error rate of 4.88%. Furthermore, the high Kappa and MCC are 0.90, with a Jaccard Index of 98.48%. The result demonstrates that the SVM-RBF classifier is outperforming for the ABC-PSO dimensionality reduction technique. Conversely, the KLDA classifier for the ABC-PSO dimensionality reduction method and the PCA-Firefly classifier for the Cuckoo Search dimensionality reduction technique demonstrated poor performance across all parameter values. This was evident from the lowest accuracy and F1 score of 53.66%, a GDR of 38.71%, and the highest error rate of 46.34%. Also, the low Kappa and MCC are 0.07, with a Jaccard Index of 36.67%. Upon analyzing individual dimensionality reduction techniques, the SVM (RBF) classifier performs better than other classifiers for the ABC-PSO DR technique, with a F1 score and GDR of 95% and a Jaccard Index of 98.48%. On the other hand, KLDA is the lowest-performing classifier due to a higher error rate of 46.34% and reduced Kappa and MCC values of 0.07. Similarly, for the CSA DR method, the linear regression classifier achieved better accuracy at 90.24%, with a strong F1 score of 90% and a lower error rate of 9.76%. Meanwhile, for the Dragon Fly DR technique, the SVM-RBF classifier attained a higher accuracy of 92.68%, a GDR of 92.50%, and a high Matthews correlation coefficient of 0.85 due to a lower error rate of 7.32%.

**Figure 16:**
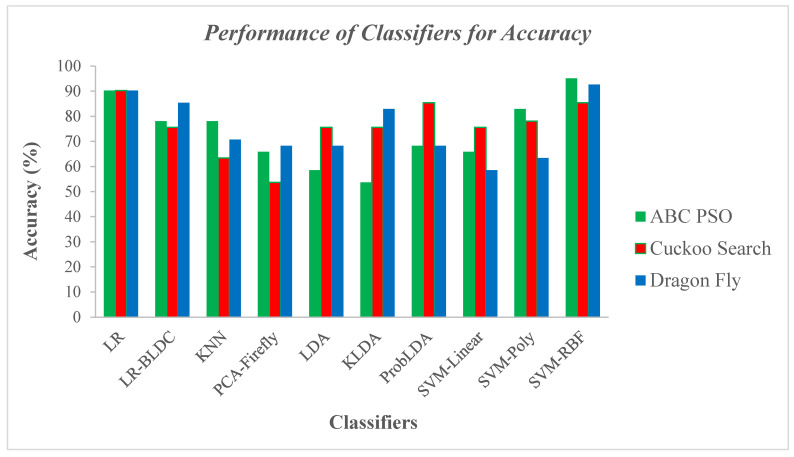
Accuracy performance across various classifiers with DR Techniques.

Figure 16 illustrates the comparison of accuracy performance among classifiers using ABC PSO, Cuckoo Search, and Dragon Fly DR techniques.

Figure 16 presents the performance evaluation of classifiers across various DR techniques based on accuracy. Figure 16 reveals that the SVM-RBF classifier reigns supreme with an accuracy of 95.12%. Conversely, the KLDA classifier yields the lowest accuracy, at 53.66% for the ABC PSO DR method. Similarly, with CSA dimensionality reduced values, the LR classifier achieves the highest accuracy of 90.24%, while PCA-Firefly exhibits the lowest accuracy at 53.66%. The SVM-RBF classifier exhibited robust performance, achieving a notable accuracy of 92.68% when paired with the Dragonfly DR method. In contrast, the SVM with linear kernel struggled comparatively, yielding a considerably lower accuracy of 58.54%. Additionally, Figure 16 shows that the LR classifier performed well across all three DR techniques, achieving an accuracy of 90.24%.

**Figure 17:**
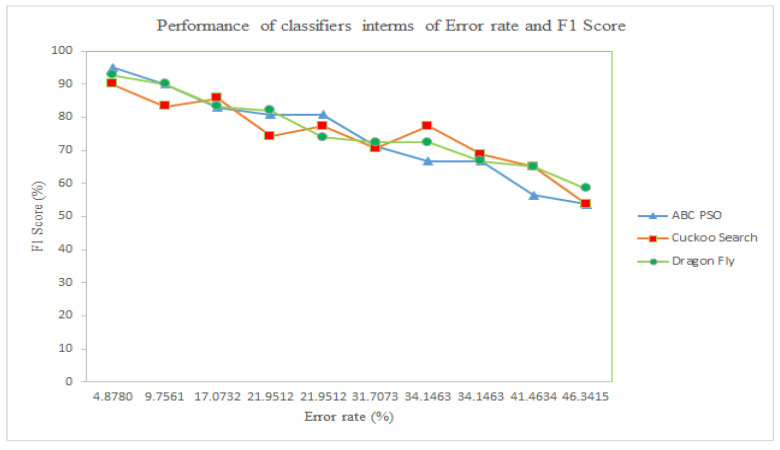
Significance of Error Rate and F1 Score Performance of Classifiers for different DR Techniques.

Figure 17 shows that all classifiers using the three different DR techniques achieve an F1 score of around 60% or higher, indicating they are performing significantly better than random guessing. This indicates a notable alignment between the model’s forecasts and the true class labels. It is also perceived that the maximum error rate observed is 46%, while the highest F1 score is 95%.

**Figure 18:**
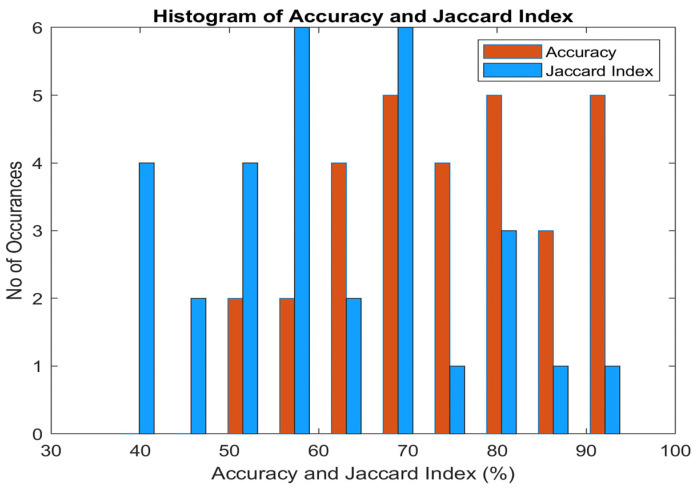
Significance of Accuracy and Jaccard Index Performance of Classifiers.

Figure 18 presents the histogram of accuracy and the Jaccard index for the classifiers. It is perceived that the maximum accuracy reaches 95%, while the highest Jaccard index is 90%. The accuracy histogram is left-skewed, indicating that the classifier’s accuracy does not drop below 50% for any of the DR techniques. The Jaccard index histogram spans the entire spectrum, with values below 50% attributed to the classifiers’ high false-positive rates.

### 5.4. Analysis of the Computational Complexity of Classifiers

Computational complexity is an important performance metric for classifiers. It is evaluated by considering the input size, denoted as m. The computational complexity remains very low when the input size is O(1). However, the computational complexity increases with the number of inputs. In this research, the computational complexity is independent of input size, which is a highly desirable characteristic for any algorithm. The term O(log⁡m) denotes the logarithmic rise in computational complexity with respect to m.

The computational complexity of classifiers across different DR techniques is presented in Table 7. From Table 7, the results clearly demonstrate that the SVM-RBF classifier has the highest computational complexity when used with the ABC-PSO and dragonfly DR techniques. Consequently, SVM-RBF achieves the highest accuracy, with 95.12% for the ABC-PSO dimensionality reduction technique and 92.68% for the dragonfly DR technique. Conversely, the KLDA classifier for the ABC-PSO dimensionality reduction technique and the PCA-Firefly classifier for the cuckoo search DR values produce the lowest accuracy of 53.66% while also having high computational complexity. This is attributed to their high false-positive rate and low Jaccard index.

### 5.5. Limitations

Table 8 compares the results of various machine learning methods used for CVD classification from PPG data. It explores the potential of using PPG data for the early identification of CVD and the prediction of associated diseases. The analysis highlighted promising classification techniques that could be beneficial for screening and identifying CVD patients. The main limitation of this work is that the PCA Firefly classifier provides the lowest average classification accuracy across all three optimization techniques, leading to an increased likelihood of false alarms in second-to-second detection of PPG signals. At the same time, 30 s segmented epochs of PPG signals were used to improve the classification accuracy of the classifiers. However, this could lead to overfitting during training, resulting in artificially high accuracy. To address this, a compromise was made by using one-minute segments of raw PPG signals to achieve more reliable classification accuracy.

As seen in Table 8, various machine learning classifiers, such as LR, NB, RBF NN, DCNN, KNN, DNN, ELM, ANN, and SVM (RBF) have been utilized for classification of CVD from clinical database. The performance spectrum of these classifiers spans from a moderate 65% to an impressive 95% in terms of accuracy. However, this study specifically targets CVD detection using Capnobase dataset, with SVM (RBF) achieving the highest accuracy of 95.12%.

## 6. Conclusions

The main objective of this work is to classify PPG signals as either normal or indicative of cardiovascular disease. High-quality PPG samples were obtained by extracting useful features using heuristic-based DR methods such as ABC-PSO, Cuckoo Search, and Dragonfly techniques. Ten classifiers were used for this purpose: LR, LR-BLDC, KNN, PCA-Firefly, LDA, KLDA, ProbLDA, SVM-Linear, SVM-Polynomial, and SVM-RBF. The SVM-RBF classifier, combined with the hybrid ABC-PSO dimensionality reduction technique, demonstrated superior performance. This approach achieved a remarkable accuracy of 95.12% while maintaining a minimal error rate of just 4.88%. Furthermore, it exhibited robust reliability, as evidenced by its high Matthews correlation coefficient and Kappa values, both reaching 0.90. Additionally, the second highest accuracy of 92.62% was achieved by the SVM-RBF classifier for Dragon Fly optimized values. The third highest accuracy of 90.24% was obtained by the LR classifier across all three DR techniques. Future research is focusing on convolutional neural networks (CNNs) and deep neural networks (DNNs) to swiftly detect cardiovascular disease.

## Figures and Tables

**Table 1:** Feature extraction and its limitations in previous related work.

S. No	Author and Year	Database	Feature Extraction	Classifiers Used	Evaluation Metric	Limitations
1	Ihsan et al. (2022) [20]	MIMIC II	Wavelet transform and time-domain features	Decision tree (DT)	Accuracy: 94.4% sensitivity: 100% specificity: 90.9%	Lack of model interpretability.
2	Pal et al. (2020) [21]	Medical College and Hospital, Kolkata.	Time domain features-Double differentiation for peak and trough identification in cardiac cycles	DT, Discriminant Analysis, Logistic Regression (LogR), SVM, KNN, Boosted Trees (BT)	For Boosted tree Accuracy = 94% Sensitivity = 95% Precision = 97%	Requires the tuning of a regularization parameter, which can be challenging, and may arbitrarily select correlated features.
3	Kanawade et al. (2019) [22]	PhysioNet MIMIC II	Crest to crest interval feature, valley to valley interval feature, transit time and beats per minute feature	ANN, SVM, LogR, DT, Random forest	SVM Accuracy = 97.67%	High computational cost for handling large datasets and a lack of a unique solution make it highly sensitive to hyperparameter settings.
4	Paradkar et al. (2017) [23]	PhysioNet MIMIC II	Temporal features obtained through wavelet Transform	SVM	Accuracy = 85% Specificity = 78%	Insufficient discussion regarding the possible biases in the dataset employed for predictive analytics.
5	Banerjee et al. (2016) [24]	MIMIC II and Inhouse datasets	Time domain features, Frequency Domain Features from HRV	SVM-RBF	Accuracy: 85% for MIMIC II and 80% for inhouse dataset	Selection of tuning parameters are challenging and Can be utilized only in clinics with limited infrastructure.
6	Xing et al. (2016) [25]	MIMIC II	Features obtained through Fast Fourier Transform	ANN	Average Accuracy: 82.33%	Resource-intensive for large feature sets and may fail to capture interactions between feature.
7	Chakraborty et al. (2020) [26]	PPG data from actual subjects using BIOPAC MP 45	Different time-plane parameters	DT, LogR, KNN SVM-linear, SVM-Nonlinear	For SVM-linear Sensitivity: 92.70% Accuracy: 95.4%	Absence of large-scale validation, the actual capabilities and limitations of the proposed system for diagnosing myocardial infarction remain certain.
8	Mangathayaru et al. (2020) [27]	BIDMC-PGG	Features obtained through dual-tree complex wavelet transform (DT-CWT)	Neural network architecture composed of successive GRU layers	Accuracy: 98.82%	Highly reliant on the selection of nearest neighbors, and may perform poorly when handling imbalanced datasets.
9	Prabhakar et al.(2019) [28]	Capnobase dataset	Chi square PDF, Density Peaks, Chi square CDF, Harmonic search, Elephant search, Particle swarm, Chicken swarm and Cat swarm optimization	LR, SVM-linear, SVM-Polynomial, SVM-Gaussian, KNN, ANN, NBC, DT, GMM, ELM	Accuracy: 99.48%	May not investigate the effects of varying hyperparameters on the performance of the proposed models, which might have impacted the overall results.
10	Tjahjadi et al. (2020) [29]	PPG-BP figshare database	Features obtained through Short-Time Fourier Transform	Bidirectional long short-term memory (BLSTM) network	Average accuracy: 96.20%	A larger and more sample size is required to further validate and refine the classification performance.

**Table 2:** Selection of parameters for heuristic algorithms.

Parameters	Heuristic Algorithms		
	ABC-PSO	CSA	DFA
Population Size	200	200	200
Control parameters	Inertia weight ω: 0.45 Acceleration coefficients $c_{1}=1$ and $c_{2}=1.1$	Probability $P_{a}$= 0.4 Step Size *α* = 1.5	Separation s=0.05 Alignment a=0.06 Cohesion c=0.06 Attraction f=0.05 Distraction e=0.2
Algorithm	Swarm intelligence with Hybrid	Levy flight	Swarm intelligence
Stopping Criteria	Training MSE of 10^−5^	Training MSE of 10^−5^	Training MSE of 10^−5^
Number of iteration	200	200	200
Local Minima Problem	Available in ABC. With proper selection of $c_{1}$ and $c_{2}$ in the PSO algorithm through trial and error method. The local minima problem will be solved.	No local minima problem	No local minima problem
Over fitting	Over fitting is available due to α and β values of ABC. This can be overcome with the proper selection of Weight (ω) of PSO Algorithm	Over fitting is not presented	Over fitting is not presented

**Table 3:** Average Statistical Metrics of ABC-PSO, Cuckoo Search, and Dragonfly Dimensionality Reduction Methods for Normal and CVD Patients.

Dimensionality Reduction Techniques	Category	Statistical Metrics						
		Mean	Variance	Skewness	Kurtosis	PCC	Sample Entropy	CCA
ABC-PSO	Normal	0.0732	0.0063	−0.1165	0.2713	−0.0597	9.9494	0.1066
	CVD	0.7872	0.3353	−0.1000	0.1435	0.0133	9.9473	
Cuckoo search	Normal	0.5236	0.0475	0.1575	−0.4556	0.3393	9.9494	0.3674
	CVD	7.8931	34.5391	−0.0901	−1.7290	0.2294	4.9919	
Dragonfly	Normal	−1.5850	378.4756	−0.0243	−0.9585	−0.2145	9.9499	0.4621
	CVD	−3.4728	271.9735	0.0381	−0.6919	0.1044	9.9522	

**Table 4:** Comparative Analysis of Training and Testing Mean Square Error Across Various Dimensionality Reduction Approaches.

Classifiers	ABC PSO		Cuckoo Search		Dragon Fly	
	Training MSE	Testing MSE	Training MSE	Testing MSE	Training MSE	Testing MSE
Linear Regression	3.52 × 10^−9^	2.92 × 10^−7^	5.69 × 10^−9^	1.37 × 10^−8^	5.99 × 10^−9^	1.44 × 10^−6^
Linear Regression with BDLC	2.32 × 10^−6^	1.10 × 10^−4^	9.69 × 10^−8^	8.65 × 10^−6^	6.03 × 10^−8^	2.72 × 10^−6^
KNN (weighted)	5.72 × 10^−8^	1.44 × 10^−6^	4.60 × 10^−6^	2.81 × 10^−3^	7.02 × 10^−8^	3.24 × 10^−6^
PCA firefly	6.65 × 10^−7^	3.80 × 10^−5^	8.45 × 10^−6^	6.08 × 10^−3^	8.69 × 10^−6^	6.25 × 10^−5^
LDA	6.69 × 10^−5^	5.48 × 10^−3^	5.05 × 10^−6^	1.44 × 10^−5^	5.54 × 10^−6^	2.70 × 10^−5^
KLDA	7.34 × 10^−6^	4.84 × 10^−3^	4.63 × 10^−8^	1.69 × 10^−6^	6.63 × 10^−6^	1.22 × 10^−5^
ProbLDA	5.83 × 10^−6^	3.06 × 10^−5^	7.97 × 10^−8^	6.76 × 10^−6^	5.99 × 10^−7^	1.68 × 10^−5^
SVM (Linear)	4.05 × 10^−6^	1.69 × 10^−4^	4.85 × 10^−8^	1.82 × 10^−6^	7.89 × 10^−6^	9.03 × 10^−3^
SVM (Polynomial)	8.29 × 10^−8^	6.76 × 10^−6^	6.74 × 10^−8^	1.44 × 10^−6^	8.20 × 10^−7^	7.29 × 10^−6^
SVM(RBF)	1.92 × 10^−6^	2.45 × 10^−9^	5.38 × 10^−7^	1.85 × 10^−5^	2.45 × 10^−10^	3.62 × 10^−9^

**Table 5:** Optimal Parameter Selection for Classifiers.

Classifiers	Optimal Parameters of the Classifiers
Linear Regression (LR)	Uniform weight w = 0.451, bias: 0.003, Criterion: MSE
LR with BLDC	The cascading configuration of LR with the following BLDC parameters: Class mean $\mu_{p}=0.8\;$and $\mu_{q}=0.1$, Prior probability *P*(*x*): 0.5
K-Nearest Neighbors (KNN)	Number of clusters = 2
PCA Firefly	PCA: A threshold value of 0.72 and decorrelated Eigen vector $w_{k}$, using a trial and error training approach Firefly: Initial conditions of $\alpha_{s}\left(0\right)$= 0.65, γ= 0.1 For both PCA and firefly, consider MSE of (10)^−5^ or reaching a maximum of 1000 iterations, whichever comes earliest. Criterion: MSE
Linear Discriminant Analysis (LDA)	Weight w = 0.56, bias: 0.0018
Kernel LDA (KLDA)	Number of clusters: 2, w1: 0.38, w2: 0.642, bias: 0.0026 ± 0.0001
Probabilistic LDA(ProbLDA)	Weight w = 0.56, bias: 0.0018, Assigned Probability > 0.5
SVM-Linear	Class weights: 0.4 Parameter for Regularization [C]: 0.85 Criteria for Convergence: MSE
SVM-Polynomial	Parameter for Regularization [C]: 0.76 Class weights: 0.5 Kernel Function Coefficient [Gamma]: 10 Criteria for Convergence: MSE
SVM-RBF	Parameter for Regularization [C]: 1 Class weights: 0.86 Kernel Function Coefficient [Gamma]: 100 Criteria for Convergence: MSE

**Table 6:** Comparative Analysis of Classifiers on Dimensionality Reduced Features.

DR Techniques	Classifiers	Accuracy (%)	GDR (%)	Error Rate (%)	Kappa	MCC	F1 Score (%)	JI (%)
ABC-PSO	Linear Regression	90.24	89.74	9.76	0.80	0.80	90.00	81.82
	LR-BLDC	78.05	72.73	21.95	0.56	0.60	80.85	67.86
	K-Nearest Neighbors	78.05	72.73	21.95	0.56	0.60	80.85	67.86
	PCA Firefly	65.85	57.58	34.15	0.32	0.32	66.67	50.00
	Linear Discriminant Analysis	58.54	48.48	41.46	0.17	0.17	56.41	39.29
	Kernel LDA	53.66	38.71	46.34	0.07	0.07	53.66	36.67
	Probabilistic LDA	68.29	59.38	31.71	0.37	0.38	71.11	55.17
	SVM-Linear	65.85	57.58	34.15	0.32	0.32	66.67	50.00
	SVM-Polynomial	82.93	81.08	17.07	0.66	0.66	82.93	70.83
	SVM-RBF	95.12	95.00	4.88	0.90	0.90	95.00	90.48
Cuckoo Search	Linear Regression	90.24	89.74	9.76	0.80	0.80	90.00	81.82
	LR-BLDC	75.61	70.59	24.39	0.51	0.52	77.27	62.96
	K-Nearest Neighbors	63.41	53.13	36.59	0.27	0.27	65.12	48.28
	PCA Firefly	53.66	38.71	46.34	0.07	0.07	53.66	36.67
	Linear Discriminant Analysis	75.61	74.36	24.39	0.51	0.53	70.59	54.55
	Kernel LDA	75.61	70.59	24.39	0.51	0.52	77.27	62.96
	Probabilistic LDA	85.37	85.00	14.63	0.71	0.72	83.33	71.43
	SVM-Linear	75.61	75.00	24.39	0.51	0.55	68.75	52.38
	SVM-Polynomial	78.05	76.92	21.95	0.56	0.58	74.29	59.09
	SVM-RBF	85.37	83.78	14.63	0.71	0.71	85.71	75.00
Dragon Fly	Linear Regression	90.24	89.74	9.76	0.80	0.80	90.00	81.82
	LR-BLDC	85.37	85.00	14.63	0.71	0.72	83.33	71.43
	K-Nearest Neighbors	70.73	62.50	29.27	0.42	0.44	73.91	58.62
	PCA Firefly	68.29	58.06	31.71	0.37	0.39	72.34	56.67
	Linear Discriminant Analysis	68.29	58.06	31.71	0.37	0.39	72.34	56.67
	Kernel LDA	82.93	81.58	17.07	0.66	0.66	82.05	69.57
	Probabilistic LDA	68.29	62.86	31.71	0.36	0.37	66.67	50.00
	SVM-Linear	58.54	46.88	41.46	0.17	0.17	58.54	41.38
	SVM-Polynomial	63.41	53.13	36.59	0.27	0.27	65.12	48.28
	SVM-RBF	92.68	92.50	7.32	0.85	0.85	92.68	86.36

**Table 7:** Computational complexity for all classifiers across different DR methods.

Classifiers	Heuristic Dimensionality Reduction Techniques		
	ABC-PSO	CSA	DFA
Linear Regression (LR)	$O(m^{5})$	$O(2m^{2}\log m)$	$O(4m^{2}\log m)$
LR with BLDC	$O(m^{7})$	$O(2m^{4}\log m)$	$O(4m^{4}\log m)$
K-Nearest Neighbors (KNN)	$O(m^{5})$	$O(2m^{2}\log m)$	$O(4m^{2}\log m)$
PCA Firefly	$O(m^{9}\log m)$	$O(2m^{3}\log2m)$	$O(3m^{3}\log2m)$
Linear Discriminant Analysis (LDA)	$O(m^{5})$	$O(2m^{2}\log m)$	$O(4m^{2}\log m)$
Kernel LDA (KLDA)	$O(m^{6})$	$O(2m^{3}\log m)$	$O(4m^{3}\log m)$
Probabilistic LDA(ProbLDA)	$O(m^{6}logm)$	$O(2m^{3}\log2m)$	$O(4m^{3}\log2m)$
SVM-Linear	$O(2m^{4}\log m)$	O(4mlog⁡m)	O(8mlog⁡2m)
SVM-Polynomial	$O(2m^{5}\log m)$	$O(4m^{2}\log2m)$	$O(8m^{2}\log2m)$
SVM-RBF	$O(2m^{6}\log4m)$	$O(2m^{3}\log5m)$	$O(8m^{3}\log5m)$

**Table 8:** Comparison of previous works on CVD classification using PPG signals.

SL.NO	Authors	Dataset	Number of Subjects	Classifiers	Classes	Accuracy (%)
1	Rajaguru et al. [51] 2023	Capnobase dataset	Single patient	LR	CVD, Normal	65.85%
2	Al Fahoum et al. [52] 2023	Internal medicine clinic of Princess Basma Hospital	200 healthy and 160 with CVD	NB	Normal and abnormal	89.37%
3	Prabhakar et al. [53] 2020	Capnobase dataset	28 CVD 14 Normal	SVM–RBF RBF NN	CVD, Normal	95.05% 94.79%
4	Liu et al. [54] 2022	GitHub https://github.com/zdzdliu/PPGArrhythmiaDetection (accessed on 9 October 2024)	45 Subjects	DCNN	CVD, Normal	85%
5	Hosseini et al. [55] 2015	Tehran Heart Center	18 Normal 30 CVD	KNN	Low risk High risk	81.5%
6	Miao and Miao [56] 2018	Cleveland Clinic Foundation	303 patients	DNN	CVD, Normal	83.67%
7	Shobita et al. [57] 2016	Biomedical Research Lab	30 healthy 30 pathological	ELM	Healthy Risk of CVD	89.33%
8	Soltane et al. [58] 2005	Seremban Hospital	114 healthy 56 pathological	ANN	CVD, Normal	94.70%
9	This research	Capnobase dataset	21 Normal 20 CVD	SVM-RBF	CVD, Normal	95.12%

LR-Linear regression; NB- Naive Bayes; DCNN-Deep Convolutional Neural Network; SVM-RBF- Support Vector Machine-Radial Basis Function; RBF NN-Radial Basis Function Neural Network; KNN- K-Nearest Neighbor; ELM- Extreme learning machine; DNN-Deep Neural Network; ANN-Artificial Neural Network.

## Data Availability

The data that support the findings of this study are available from the corresponding author upon reasonable request.
